# Risk allocation in a freshwater gastropod

**DOI:** 10.1093/beheco/araf078

**Published:** 2025-07-13

**Authors:** Denis Meuthen

**Affiliations:** Evolutionary Biology, Bielefeld University, Konsequenz 45, D-33615 Bielefeld, Germany

**Keywords:** predation risk, risk allocation, threat-sensitivity, crawl-out behavior, *Physa acuta*, *Physella acuta*

## Abstract

To balance the basic needs of organisms, internal and external cues are used to inform the optimal behavioral strategy. Some of the best-studied related cognitive rules have emerged in predator-prey contexts, such as the threat-sensitivity hypothesis, which postulates that prey should adjust their antipredator behavior in accordance with the level of risk. Extending this theory, the risk allocation hypothesis posits that under long-term sustained high predation risk, individuals should decrease their antipredator responses towards risky stimuli so as to meet their energetic demands. Evidence for the risk allocation hypothesis has been mixed in invertebrates, particularly in gastropods, which are classic model systems for antipredator responses. This may be due to past studies frequently lacking sham controls and/or sufficient certainty about the risk regime. The present study in the aquatic gastropod *Physella acuta* controls for these factors by crossing long-term background risk, ie lifelong consistent exposure to conspecific alarm cues, which reliably signal high predation risk (high-risk), or a water control (low-risk) with exposure to a high-risk or low-risk stimulus. Crawl-out behavior is an adaptive antipredator response in gastropods. In accordance with threat-sensitivity, high-risk stimuli induced increased crawl-out behavior independent of background risk. Providing partial support for risk allocation, high background risk induced lower responsivity to both low-risk and high-risk chemical stimuli. This may be because cue addition also provided tactile cues that could be considered risky by high background risk snails. Altogether, the present well-controlled research contributes novel data to the hitherto mixed evidence for risk allocation in gastropods.

## Teaser

Snails have to trade off their time and energy investment between foraging and responding to cues of predation as they can’t do both at the same time. We exposed aquatic snails from birth onwards to either low or high perceived risk. Afterwards, we found that low-risk individuals respond to risk-related stimuli but high-risk individuals do not. Thus, long-term high risk causes snails to decrease their antipredator responses towards stimuli that are considered risky so as to meet their foraging needs.

## Introduction

To maximize their fitness, organisms need to balance their basic needs such as growth, survival and reproduction. To achieve this feat, they have to obtain and integrate environmental information (McNamara and Houston 1986). In the following process of decision-making, the optimal behavioral strategy for an individual organism is then derived from this input (Blumstein and Bouskila 1996; Budaev et al. 2019). The ability to do so has strong adaptive significance (Suddendorf et al. 2018), hence even unicellular organisms are capable of this feat (Lyon 2015).

One of the best-studied decision-making processes in nature is prey behavior in a predation context (Lima and Dill 1990; Lima 1998). Predation is one of the most forceful selection pressures in nature (Lima and Dill 1990) that fluctuates temporally and spatially (Sih 1992; Sih et al. 2000). While prey responses to predation risk increase survival, they are also energetically costly and the time invested in the responses conflict with alternate fitness-related activities such as foraging or searching for mates (FitzGibbon 1994; Teplitsky et al. 2005; Slos et al. 2009). Consequently, prey animals have evolved several cognitive rules to optimize their decision-making.

A well-known cognitive rule is called the threat-sensitivity hypothesis. It postulates that prey should adjust their antipredator behavior in accordance with the risk posed by predators (Helfman 1989). In other words, increased predation risk should be met with a positively correlated level of antipredator behavior. Previous empirical tests demonstrate the ubiquity of this hypothesis across diverse taxa (fish: Helfman (1989), gastropods: Rochette et al. (1997), crustaceans: Wood and Moore (2020), amphibians: Ferrari and Chivers (2010), insects: Roux et al. (2014)). The risk allocation hypothesis is another cognitive rule that is based on the threat-sensitivity hypothesis but also has a counterintuitive prediction. That is, in environments where risk is continuously high, prey should display lower than expected antipredator behavior during risky periods to meet their foraging demands (Lima and Bednekoff 1999). While this hypothesis has been also tested across diverse taxa, results have been inconsistent (Ferrari et al. 2009). This is frequently hypothesized to be due to suboptimal experimental design, for example due to the use of methods that violate core assumptions of risk allocation (Ferrari et al. 2009). One of these is that prey must have perfect information about the risk regime (Ferrari et al. 2009), as imperfect information may reduce risk allocation in prey that have evolved to use perfect information (Luttbeg 2017).

While following tests of the risk allocation hypotheses in vertebrates have benefited from considering these core assumptions alongside careful experimental design as it made measurable behavioral responses more consistent (eg Meuthen et al. 2019), invertebrates have been neglected in that respect. In particular, well-controlled tests of risk allocation are rare in gastropods, a classic taxon for the study of antipredator responses (Hoverman 2010) with great ecological (Mas‐Coma et al. 2009; Colley et al. 2014) and economic importance (Morris et al. 2019; Wijsman et al. 2019). Unsurprisingly, in this taxon, support for the risk allocation hypothesis remains mixed to date. In the marine snail *Littorina* sp., Hamilton and Heithaus (2001) found partial support for the risk allocation hypothesis as with longer periods of perceived high risk, snails increased foraging in high-risk periods. However, in low-risk periods, this relationship was absent. In another marine snail *Nucella lapillus*, Trussell et al. (2011) did not find evidence for the risk allocation hypothesis. Later, Matassa and Trussell (2014) found partial support for the risk allocation hypothesis in the same species only when individuals were starved. That is because in contrast to the predictions of this hypothesis, foraging under safety did not increase with increasing percentage of time spent under high risk. In yet another study on the same species, Donelan and Trussell (2018) found no support for the risk allocation hypothesis as embryonic high risk induced more behavioral defenses in the absence of risk and did not alter responses towards high-risk stimuli. In contrast, Sih and McCarthy (2002) provided support for the risk allocation hypothesis with the freshwater snail *Physa gyrina*. In another physid snail, *Physa pomilia,*Salice and Plautz (2011) found that offspring from wild-caught individuals (ie those exposed to high risk) became less responsive to repeated high-risk stimuli whereas offspring from lab-reared individuals (ie those exposed to low risk) did not. Under low risk, both populations behaved similarly. In yet another physid species, *Physa acuta*, Beaty et al. (2016) found support for the risk allocation hypothesis as in the absence of risk, high-risk individuals were less likely to show antipredator behavior, and high-risk individuals were also less responsive to high-risk cues. In the same species, Tariel et al. (2020a) also found lower responsiveness of high-risk individuals to risky stimuli, but previous risk exposure did not affect behavioral defenses under low risk (ie no evidence for more foraging behavior in safety for high-risk individuals).

This inconsistency between these gastropod studies may arise from not only suboptimal experimental design such as the absence of proper sham control stimuli during sampling (Salice and Plautz 2011; Beaty et al. 2016), but also due to the fact that only few studies satisfy the core assumption of the risk allocation hypothesis: that prey must have perfect information about the risk regime (Ferrari et al. 2009; Luttbeg 2017). Imperfect information about risk may arise from risk exposure periods that do not extend beyond the embryonic stage (Donelan and Trussell 2018) or beyond 2 to 3 d in adulthood (Hamilton and Heithaus 2001; Sih and McCarthy 2002). Furthermore, uncertainty about risk may arise from repeated testing of the same individuals in different risk environments (Tariel et al. 2020a). Another source of uncertainty about risk may arise from the fact that different chemical cues indicating predation risk differ in their information content (Wisenden 2015). First, alarm cues released from damaged conspecifics, known as conspecific alarm cues, are innately recognized and typically induce standard antipredator responses. In contrast, the risk associated with predator odors that are released independent of prey consumption, ie kairomones, is not always innately recognized and has to be learned when predator species are unfamiliar (Batabyal and Lukowiak 2023). In addition, kairomones are known to induce predator-specific responses (Lakowitz et al. 2008), which is concerning for tests of risk allocation as predator-specific patterns of foraging may explain variation in prey responses. Many of the abovementioned studies mainly manipulated risk through exposure to caged predators that release kairomones. While Hamilton and Heithaus (2001) used kairomones from caged crustacean predators as risk stimuli exclusively, Trussell et al. (2011), Matassa and Trussell (2014), Beaty et al. (2016) and Donelan and Trussell (2018) also provided caged predators with snails as food. However, they did not determine whether snails have actually been damaged or eaten, ie whether conspecific alarm cues have actually been released. In contrast, Sih and McCarthy (2002), Salice and Plautz (2011) and Tariel et al. (2020a) also added crushed conspecifics to crustacean kairomones to ascertain the concurrent presence of conspecific alarm cues. At first sight, this appears to be the best approach as DeWitt et al. (1999) suggested that snails display their full antipredator response when exposed to cues from both predators and crushed conspecifics. However, it still remains possible that this approach induces predator-specific responses. Taken together, we suggest that using conspecific alarm cues alone, which avoids experience-dependent and predator-specific variation in risk estimation, may be better suited for conveying perfect information about the risk regime to prey. Thus, here we test the risk allocation hypothesis in gastropods by exposing prey to conspecific alarm cues (a reliable indicator of risk) for a long time period (continuously from birth onwards over 28 d), which should satisfy the core assumption of the risk allocation hypothesis: that prey must have perfect information about the risk regime (Ferrari et al. 2009; Luttbeg 2017). To this end, we use the classic model system *Physella acuta* (Draparnaud 1805), a physid freshwater snail with global distribution due to its invasive potential (Vinarski 2017). This species is well-studied in regard to behavioral antipredator responses (Turner 1996; DeWitt et al. 1999; Kain and McCoy 2016), including the risk allocation hypothesis (Beaty et al. 2016; Tariel et al. 2020a). Upon a single stimulus of high perceived predation risk, *P. acuta* increases its crawl-out behavior whereby it reaches a a safe refuge outside of the water (Covich et al. 1994; Tariel et al. 2020a, 2020b; Tariel-Adam et al. 2023). In our study, we raise *P. acuta* under either high or low background risk (ie consistent exposure to either conspecific alarm cues or a water control) and then, study their crawl out before (baseline) and in a fully crossed 2 × 2 design, after exposure to either a high-risk (conspecific alarm cue) or a low-risk (water control) stimulus. First, following the threat-sensitivity hypothesis and previous papers, we hypothesize that a high-risk stimulus should increase crawl-out behavior. Second, following the risk allocation hypothesis, high-risk snails should be less responsive to risky stimuli than low-risk individuals.

## Materials and methods

### Experimental animals

Experiments were run from January to March 2024. *P. acuta* were derived from a laboratory population whose ancestors had been collected from 10 wild populations across Europe. Ancestral *P. acuta* were collected in 2020, 2021 and 2022 across freshwater bodies in Germany, the Netherlands, France and Austria (46°48’53.2“N 13°30’22.9”E, 52°23’10.4”N 9°42’19.7”E, 52°06’58.1”N 11°38’55.0”E, 52°03’09.8”N 8°33’25.2”E, 50°43’52.1”N 7°04’21.7”E, 51°21’42.6”N 6°46’35.5”E, 45°48’46.6”N 4°57’53.6”E, 52°21’36.3”N 4°50’43.0”E, 52°21’20.2”N 4°51’35.9”E, 48°52’49.0”N 2°22’54.8”E). After transportation to the lab, we subjected them to a 5-day antibiotic treatment (ie 25 mgL^-1^ erythromycin), which has been previously shown to increase both *P. acuta* survival (DeWitt and Prestridge 2022) and their capability to produce antipredator defenses (Meuthen and Reinhold 2023). Then, individuals were crossed in a fully factorial breeding design over eight generations (alternating between outbreeding and inbreeding to ensure full crossover of alleles). Ninth-generation offspring containing alleles from eight populations each were then placed in ten replicate 20 x 30 x 20 cm (L x W x H) tanks containing 3.2 L of remineralized osmosis water, 1 g cuttlefish shell and 4 g dried mixed leaf litter (44 individuals per replicate tank, one representative snail from each genetic cross of the 8 populations over 9 generations) in August 2023 and could freely intermix (two *ad libitum* feedings per week, one 50% water change per month, leaves and cuttlefish shells were replaced) until we started breeding in January 2024.

To generate the experimental animals, we collected 114 adult snails (7 to 31 individuals per replicate tank, mean ± SD shell height: 8.39 ± 1.00 mm, shell width: 4.83 ± 0.51 mm, weight: 0.081 ± 0.027 g) and isolated them within 60 × 45 × 50 mm tanks (Boite LAB 1, Multiroir, Périgny-sur-Yerres, France). Each tank was filled with 50 ml of osmosis water, which has been remineralized to 500 ± 10 µS using a mineral powder that increases both total hardness and carbonate hardness at a 1:0.5 ratio and also contains necessary trace elements (SaltyShrimp ShrimpMineral GH/KH+, Garnelenhaus, Barsbüttel, Germany). We also supplied a 2 × 2 cm piece of floating polystyrene to facilitate egg deposition. Temperature was kept at 25 ± 1 °C, and light was provided in a 12:12 cycle (from 8am to 8pm) from 27 cm above the waterline of each tank by daylight (5500K) LED strips (LFL-40W2-004, Bioledex, Augsburg, Germany) that were dimmed to 25% of their maximum output with a transformator (APV-35-12, MeanWell, New Taipei City, Taiwan). In addition, each tank was covered by a transparent lid made from the same material as the tank itself. Every day, parents received a full water change and were fed with ad libitum crushed algae tabs (Spirulina Tabs Nature, sera, Heinsberg, Germany, n = 20, mean ± SD of provided mass 0.027 ± 0.004 g) along with ad libitum crushed sepia shells (n = 20, mean ± SD of provided mass 0.044 ± 0.006 g). 4 d later, we removed the parents from the breeding tank. Afterwards, we discarded infertile clutches as indicated by non-developing embryos, and 1 d before hatching (*P. acuta* eggs hatch on day 7 after deposition) we used tweezers under a stereo microscope to split each clutch into two halves with an equal number of eggs and placed each half into a tank with 50 ml fresh water of the same kind as before along with ad libitum (ie 0.044 g) crushed sepia shells. In total, we successfully collected clutches from 22 parental individuals and generated 22 offspring families.

Offspring tanks were kept at the same light and temperature conditions as parental tanks. The two halves from each clutch were haphazardly assigned to one of the two background treatments: a) fresh water, b) conspecific alarm cues. From hatching onwards, three times per week with at least one intermission day each, offspring received a full water change, ad libitum food (each n = 20, mean ± SD of provided mass; 0 to 6 d of age: 0.003 ± 0.002 g; 7 to 14 d of age: 0.007 ± 0.001 g; 14 to 20 d of age: 0.027 ± 0.004 g; 21 to 28 d of age: 0.072 ± 0.008 g) and sepia (0.044 g) was provided. Afterwards, they received their assigned background treatment (12.5 µl of a water control or 12.5 µl of a liquid containing 1 crushed snail/ml, which is equivalent to 1 crushed snail in 4 L of water), a standard concentration known to elicit strong antipredator responses across different *P. acuta* studies (Tariel et al. 2020a; Meuthen and Reinhold 2023). As the *P. acuta* alarm cue putatively decays after 41 h (Turner and Montgomery 2003), this exposure regimen constitutes a 6-day continuous exposure period per week and as such, has been established as a standard in multiple previous studies (Auld and Relyea 2010, 2011; Meuthen and Reinhold 2023) to ensure constant exposure to these cues.

As in gastropods, density effects impact growth and behavior (Dan and Bailey 1982; Conner et al. 2008), following established practices aiming to minimize density effects in *P. acuta* (Beaty et al. 2016; Goeppner et al. 2020), 14 d after hatching, available individuals were separated into groups of six within new tanks of the same dimensions and contents as before. In total, we set up n = 16 tanks per treatment. This sample size was based on a power analysis on an unpublished preliminary study (n = 20 tanks, ie 120 individuals) on the effect size of high-risk and low-risk stimulus treatments on the change in the proportion of snails above the waterline. The estimated effect size of stimulus risk was d_Cohen_ = 1.23, 95% CI [0.66, 1.79]. The required sample size according to the estimated effect size assuming 80% power and a p-value of 0.05 is n = 12 per treatment. To account for possible unexpected mortality, we instead set up a slightly larger number of tanks. Our 16 tanks per treatment resulted in a total number of 64 tanks containing 385 individuals. We raised 385 instead of 384 individuals (assuming 6 snails per tank in 64 tanks) as in one instance, we found that we accidentally raised seven snails instead of six in a single tank, but as we calculated behavioral responses as proportions relative to available snails (see below), we decided to keep this sample in our dataset.

While we observed some mortality over the raising period, we found no statistical evidence for it to differ between background risk treatments (see §1 of the supplementary material). At an age of 28 d, all 64 tanks containing the surviving 373 individuals (high background risk & high stimulus risk: 16 tanks containing 14 different families; high background risk & low stimulus risk: 17 tanks containing 13 families; low background risk & high stimulus risk: 16 tanks containing 12 families; low background risk & low stimulus risk: 15 tanks containing 14 families) were sampled in behavioral trials.

### Chemical cue preparation

Conspecific alarm cues for background treatments were obtained from 20 adult *P. acuta* (two pools containing 10 individuals each—each pool contained one individual per replicate tank, overall mean ± SD shell height: 7.32 ± 0.69 mm, shell width: 4.24 ± 0.49 mm, weight: 0.054 ± 0.016 g) of the same population. We first measured donor shell length and width to the nearest 0.01 mm with a digital caliper (Model ABS-AOS 500-181-30, Mitutoyo, Kawasaki, Japan), and assessed their total weight (including the shell) to an accuracy of 0.1 mg on a digital scale (Quintix 124-1S, Sartorius, Göttingen, Germany). We then added 3 to 4 snails along with 500 µl of remineralized osmosis water into an 1.5 ml Eppendorff tube and crushed donors with a pellet pestle (Disposable pellet pestle, PP, Th. Geyer, Renningen, Germany) before repeating the same procedure for the next snails until all donors of a pool were crushed. We then filtered the homogenate of each pool with filter floss and diluted the extract to a total volume of 10 ml. The protocol for water controls remained the same, ie they were sham-crushed and sham-filtered to control for possible contaminants. Both alarm cues and water controls were then frozen in 0.5 ml aliquots at -20°C until use, a procedure to retain long-term efficacy of alarm cues as established in teleost fish (Lawrence and Smith 1989) and *P. acuta* (Meuthen and Mutingwende 2025). On the day of use, we thawed the cues and injected 12.5 ml into each tank; due to the 50 ml tank volume, the alarm cue concentration within tanks is equivalent to 1 snail diluted in 4 L of water, a standard concentration known to elicit strong antipredator responses across different *P. acuta* studies (Tariel et al. 2020a; Meuthen and Reinhold 2023).

To avoid habituation of experimental animals to alarm cues from specific donor individuals or to a specific batch of water control, stimulus treatment cues were prepared separately. Stimulus alarm cues were obtained from a different pool of 10 adult *P. acuta* (one individual per replicate tank, mean ± SD shell height: 7.83 ± 0.76 mm, shell width: 4.54 ± 0.39 mm, weight: 0.063 ± 0.016 g) of the same population. Alongside stimulus treatment water controls, these cues were prepared the same way as background treatment alarm cues.

### Sampling procedure

Snails were sampled in their housing tanks (dimensions: 60 × 45 × 50 mm). For sampling, tanks from each background treatment were in a fully crossed 2 × 2 design assigned to one of two stimulus treatments, a) fresh water, b) conspecific alarm cues. For sampling, tanks containing snails were moved to a different illuminated shelf and after a following 10-minute acclimation time, a 30-minute prestimulus period began. During this period, every 5 min, we counted the number of snails outside of the water, ie the number of individuals that did not touch the water with any part of the body. This measurement has been found to be the most reliable proxy for crawl-out behavior (Meuthen and Mutingwende 2025). After the 30-minute prestimulus period, we removed the lid, added the stimulus treatment (12.5 µl of a water control or alarm cues of the same concentration as before), and closed the lid again. Snails located outside of the water were not placed back in the water as touching them would constitute an even riskier stimulus. This is not an issue as snails consistently moved across the water surface (excluding the instances where all snails were inside the water, on average 0.9 snails crossed the surface within each 5-min prestimulus interval), and within snails that received a high-risk stimulus, we also found no statistical evidence for the presence of a correlation between the number of individuals outside of the water immediately prior to the addition of the stimulus and the behavioral response towards the stimulus (Spearman’s rank correlation, ρ = −0.274, S = 6952.8, p = 0.129; LRT, Χ² = 1.092, p = 0.296).

Immediately afterwards, a 30-minute poststimulus period started, during which we, again, counted the number of individuals above the water in 5-minute intervals. Hence, we collected 12 behavioral datapoints per tank, in total 768 datapoints across 64 tanks. After the trials finished, we also counted the number of snails in each tank.

As in group-based trials, the average size of individuals as well as the variation in size between individuals can influence behavioral responses (Meuthen et al. 2016, 2019, 2021), immediately following behavioral trials, we assessed the size of 248 individuals within a subset of experimental tanks (42 tanks; high background risk & high stimulus risk: 11 tanks; high background risk & low stimulus risk: 10 tanks; low background risk & high stimulus risk: 9 tanks; low background risk & low stimulus risk: 12 tanks). Due to logistic constraints, we unfortunately did not have the resources to assess the size of all 373 individuals, but ensured equal representation of each family by sampling one tank per family and background treatment. To assess individual sizes, we blotted individual snails dry with paper towels, placed them on a ~1mm² piece of play dough to ensure that their aperture opening was parallel to the camera and photographed them, aperture upwards, on top of a size standard, at 2048 × 1536 pixel resolution with a Moticam 1080 attached to a SMZ-171 stereomicroscope (Motic, Xiamen, China).

### Data analysis

#### Photographs

To obtain estimates of individual size, we assessed shell centroid size, a robust proxy for shell volume in gastropods (Osborne and Stehman 2022), using geometric morphometrics (Zelditch et al. 2012). With tpsDig2 v.2.30 (Rohlf 2015) after size calibration, we placed 11 established (Beaty et al. 2016) landmarks along with 21 semilandmarks on each photograph. Centroid size (ie the square root of the sum of distances squared from each landmark to the centroid) was then obtained after Procrustes superimposition using the geomorph R package v.4.0.7 (Adams et al. 2021). For each tank, we then calculated average snail size (ie mean centroid size) as well as coefficients of variation as proxy for snail homogeneity within tanks (CV, ie dividing the standard deviation by the mean). We did not find statistical evidence for our background treatment to affect average snail size or snail homogeneity (see §1 of the supplementary material).

### Statistical analysis

All analyses were conducted using R 4.4.0 (R Core Team 2024).

To determine to what extent the response to the stimulus depends on background and stimulus treatments, we constructed a binomial generalized linear mixed-effect model (GLMM) using the lme4 R package v.1.1-35.3 (Bates et al. 2015). Here, we entered the proportion of snails outside of the water per tank (ie the combination between the number of snails outside vs. inside the water) as the dependent variable. Fixed effects were background treatment (high-risk/low-risk), stimulus treatment (high-risk/low-risk), phase (prestimulus/poststimulus) and interval (a continuous variable representing the minute of sampling within phases) as well as all two-way, three-way and four-way interactions between these factors. We also entered interval as a random slope to account for our repeated-measures design as well as tank identity nested in family as random intercepts. In this model, we entered intervals *within* phases rather than *across* phases to control for effects of experimental time as the effect of the latter is correlated with the effect of the stimulus; preliminary analyses also revealed that our results do not change qualitatively or quantitatively when using intervals *across* phases instead.

Full models were reduced by sequentially removing non-significant (p > 0.05) interactions to obtain final models for reliable effect estimates (Engqvist 2005), but as all of our fixed effects were considered meaningful, they were retained in final models as this avoids pseudoreplication asssociated with test-qualified pooling (Colegrave and Ruxton 2017). We ascertained that none of our binomial models was overdispersed with the performance R package v. 0.12.2 (Lüdecke et al. 2024b). Statistical inferences were drawn with the use of Likelihood Ratio Tests (LRTs), ie we assessed whether the removal of variables caused significant decreases in model fits according to the Akaike information criterion; our p-values thus reflect the increase in deviance upon the removal of a variable. Model summaries shown in supplementary tables were generated with the sjPlot R package v. 2.8.16 (Lüdecke et al. 2024a). Marginal effects were extracted from final models with the emmeans R package v.1.10.1 (Lenth et al. 2020), and standardized effect sizes (d_Cohen_) alongside their 95% confidence intervals were calculated with the effectsize R package v.0.8.9 (Ben-Shachar et al. 2020). Raw means (y_Raw_) and standard deviations (SD) for each effect, converted to percentages (%), are provided alongside the results.

## Results

**Fig. 1.**
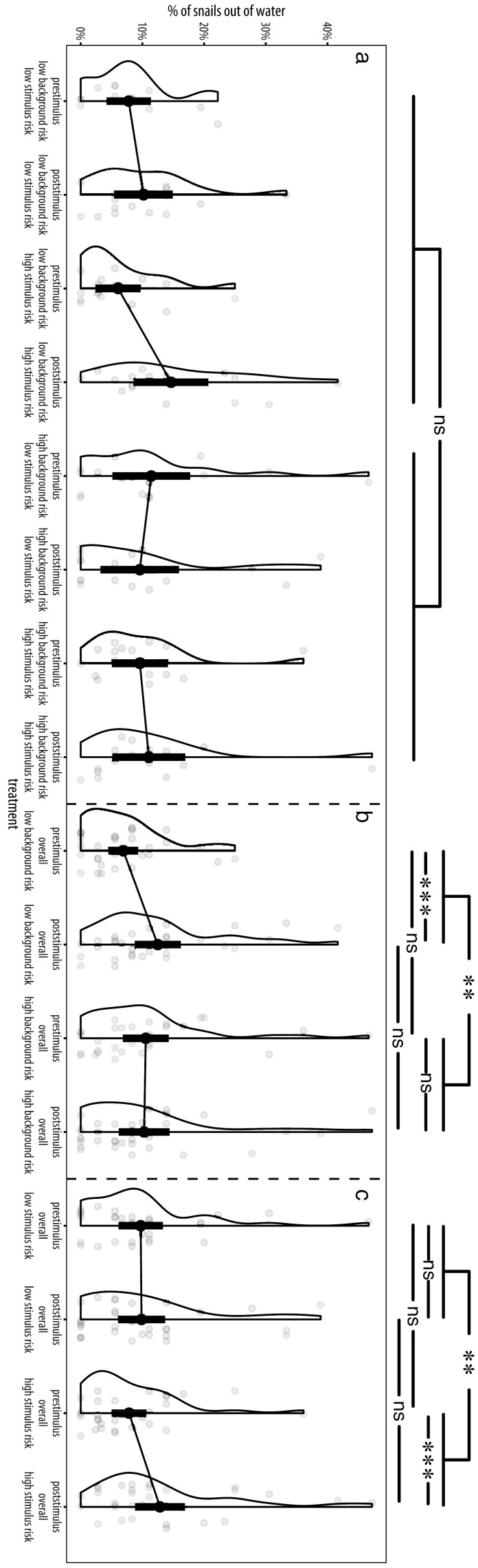
Impact of both background risk and stimulus risk on prestimulus and poststimulus crawl-out behavior (black dots and bars reflect means ± 95% confidence intervals, violin plots showcase data distribution, gray dots highlight individual datapoints) of 28-day old *Physella acuta*. In a full-factorial 2 × 2 design, individuals were lifelong consistently raised under either high background risk (conspecific alarm cues) or low background risk (water control). Afterwards, they were observed in the absence of cues during a 30-min pre-stimulus period, after which they received either a high-risk stimulus (conspecific alarm cues) or a low-risk stimulus (water control). This was followed by a 30-min post-stimulus period. Shown are a) the background risk × stimulus risk × phase interaction, b) the background risk × phase interaction and c) the stimulus risk × phase interaction. Each datapoint represents the average proportion of snails outside of the water over a 30-minute period. Lines connect prestimulus and poststimulus periods from the same background/stimulus risk treatment combination. Data were obtained from 33 high-risk and 31 low-risk tanks containing 192 and 181 individuals, respectively. Asterisks above bars visualize the significance of the interactions (topmost lines) and of differences between individual risk treatment-phase combinations, respectively. *** p < 0.001; ** p < 0.01; ns p > 0.1.

We did not find evidence for the presence of a four-way interaction (background treatment × stimulus treatment × phase × interval interaction, estimated interaction effect: 0.063, 95% CI [0.005, 0.121], Χ² = 1.625, p = 0.202), a three-way interaction (background treatment × stimulus treatment × phase interaction, estimated interaction effect: estimated interaction effect: 0.426 [−0.089, 0.926], Χ² = 0.990, p = 0.320, Fig. 1a), and many other interactions (Table S1). However, in our final models, we observed that both background risk (background treatment × phase interaction, estimated interaction effect: −0.681 [−1.047, −0.261], Χ² = 10.371, p = 0.001, Fig. 1b) and stimulus risk (stimulus treatment × phase interaction, estimated interaction effect: 0.528 [0.108, 0.924], Χ² = 6.226, p = 0.013, Fig. 1c) influenced how individuals adjusted their crawl-out behavior in response to the stimulus. In contrast, interval (ie experimental time) did not affect crawl-out behavior in a statistically significant way (estimated slope: 0.002 [−0.015, 0.018], d_Cohen_ = 0.015 [−0.127, 0.156], Χ² = 0.036, p = 0.849, Fig. S1, S2).

Post-hoc investigation disentangling the background treatment × phase interaction shows that regardless of whether low-risk or high-risk stimuli were used, snails raised in low background risk strongly responded to the stimulus as indicated by about twice as many individuals outside of the water in the poststimulus period (y_Raw_ = 12.5 ± 16.2 %) compared to the prestimulus phase (y_Raw_ = 6.9 ± 11.8 %; estimated difference: 0.672 [0.374, 0.97], d_Cohen_ = 0.458 [0.255, 0.662], z = 10.371, p < 0.001, Fig. 1b). In contrast, snails from a high background risk environment displayed similar crawl-out behavior in the prestimulus (y_Raw_ = 10.5 ± 15.3 %) and poststimulus period (y_Raw_ = 10.3 ± 16.0 %; estimated difference: −0.008 [−0.287, 0.27], d_Cohen_ = −0.006 [−0.203, 0.191], z = 10.371, p = 0.953, Fig. 1b) irrespective of the risk level of the stimulus cue. Snails from different background risk treatments did not display statistically different crawl-out behavior during either the prestimulus (estimated difference: 0.366 [−0.113, 0.845], d_Cohen_ = 0.153 [−0.047, 0.353], z = 10.371, p = 0.134) or the poststimulus period (estimated difference: −0.315 [−0.77, 0.141], d_Cohen_ = −0.138 [−0.338, 0.062], z = 10.371, p = 0.176).

Further post-hoc analysis to disentangle the stimulus treatment × phase interaction also revealed that independent of whether the background risk level was low or high, high stimulus risk also induced a clear, albeit smaller increase in the crawl-out response from the prestimulus (y_Raw_ = 7.8 ± 13.1%) to the poststimulus period (y_Raw_ = 12.8 ± 16.6 %; estimated difference: 0.596 [0.308, 0.884], d_Cohen_ = 0.414 [0.213, 0.614], z = 6.226, p < 0.001, Fig. 1c). This was not the case when stimulus risk was low as in this instance, regardless of background risk, prestimulus crawl-out behavior (y_Raw_ = 9.7 ± 14.6 %) did not differ from poststimulus behavior (y_Raw_ = 9.9 ± 15.6 %) in a statistically significant way (estimated difference: 0.068 [−0.22, 0.356], d_Cohen_ = 0.047 [−0.153, 0.247], z = 6.226, p = 0.644, Fig. 1c). These results are largely attributable to behavioral changes observable within individual groups as on average, crawl-out behavior from snails that received different stimulus treatments did not differ in a statistically significant way during either the prestimulus (estimated difference: −0.145 [−0.622, 0.331], d_Cohen_ = −0.061 [−0.261, 0.139], z = 6.226, p = 0.550) or poststimulus period (estimated difference: 0.382 [−0.073, 0.838], d_Cohen_ = 0.168 [−0.032, 0.368], z = 6.226, p = 0.100, Fig. 1c).

Controlling for variation in average snail size and variation in size within tanks within a reduced dataset did not lead to quantitatively different results (see §2 and Table S2 of the supplementary material).

## Discussion

Our results suggest that the decision-making in *P. acuta* following lifelong exposure to either low or high predation risk is in accordance with the threat-sensitivity hypothesis and also provides partial support for the risk allocation hypothesis. First, we did not find evidence for a three-way interaction between background risk, stimulus risk and phase (ie prestimulus/poststimulus), which would have indicated that individuals experiencing different background risk respond differently to high-risk and low-risk stimuli. Instead, irrespective of background risk, a high-risk stimulus induced greater crawl-out behavior than a low-risk one. Furthermore, irrespective of whether low-risk or high-risk stimuli were used, high background risk induced lower responsivity to stimuli. Lastly, average individual size and snail homogeneity in size as obtained from a subset of our data appear to be unrelated to our background risk treatment and we also did not find evidence for them to affect individual decision-making rules.

Despite us finding evidence for both high background risk and high stimulus risk to adaptively increase crawl-out behavior in *P. acuta*, the lack of a three-way interaction suggests that our study does not fully support the risk allocation hypothesis. That is because this hypothesis predicts that under continuous high background risk, prey animals need to display lower than expected antipredator behavior towards *risky* stimuli to satisfy their metabolic demands (Lima and Bednekoff 1999). In contrast, we did not find statistical evidence for individuals from different background risk levels to differ in their behavioral response towards high- and low-risk stimuli. However, our low-risk stimulus, despite just being a water injection, also involves opening and closing the lid of the tank, a tactile (vibrational) cue. While we lack knowledge on how *P. acuta* assesses risk based on tactile cues, research on *Nucella lapillus*, another gastropod species, suggests that tactile cues do communicate predation risk in gastropods (Boudreau et al. 2018). Similarly, the fact that we did not find evidence for a three-way interaction may suggest that the tactile cues that arise from stimulus addition do communicate risk in *P. acuta* as well. Given this interpretation, high background risk individuals likewise may estimate the presence of the tactile cue during a low-risk stimulus as sufficient high-risk information to respond with patterns of risk allocation. This is why we suggest that our study still provides at least partial support for the risk allocation hypothesis. Given that the present study ensured that prey have perfect information about the risk regime, a pre-requisite for testing the validity of the risk allocation hypothesis (Ferrari et al. 2009; Luttbeg 2017), and also includes proper sham controls, it is a valuable addition to previous studies on risk allocation in physid snails and other gastropods. More generally, our study also contributes a further piece of evidence to the hitherto mixed evidence for the generality of the risk allocation hypothesis across taxa (Ferrari et al. 2009).

Alternative explanations for the lack of full support for the risk allocation hypothesis may be first that despite the use of 373 experimental individuals, due to us sampling groups of snails, our real sample size is still relatively small with n = 64. Interaction power analyses suggest that at an α of 0.05, with our sample size, we had 80% power to observe at least a regression coefficient of b = 0.374 (ie medium three-way interaction effects). Of course, a true three-way interaction effect pertaining to the risk allocation hypothesis may be even smaller, or the absence of a three-way interaction may also be a Type II error. However, given that our sample sizes aligned with previous risk allocation studies in gastropods, the observed absence of a three-way interaction is more likely due to the biological explanation outlined above. Another cause may be that we did not sufficiently energy-limit snails as we kept them under ad libitum food conditions throughout the study and sufficient energy-limitation is suggested to be a pre-requisite for testing the validity of the risk allocation hypothesis (Ferrari et al. 2009). Supporting this theory is that in another gastropod, *Nucella lapillus*, risk allocation could only be observed when individuals were starved (Matassa and Trussell 2014). Yet another explanation could be that risk allocation could evolve only in populations or species that experienced constant levels of high risk over evolutionary history (Meuthen et al. 2019). Supporting this idea is that in the gastropod *P. pomilia*, Salice and Plautz (2011) found support for risk allocation only in offspring from wild-caught but not in offspring from lab-reared individuals. On one hand, to minimize inbreeding effects, here we used an artificial population constructed by crossing 10 natural populations, which may differ in their extent of evolved antipredator plasticity including risk allocation and thus give rise to a population with on average small antipredator responses. This idea is supported by the rather small effect sizes that we observed in our population when it comes to stimulus-induced increased crawl-out behavior: at maximum ~10% more snails out of the water in response to a high-risk stimulus. In comparison, Covich et al. (1994) suggest that risk induces an up to 16% increase in snails out of the water. On the other hand, Luttbeg (2017) suggests that at least theoretically, population origin should play only a minor role in explaining variation in patterns of risk allocation.

Our data further provides evidence against possible explanations for the observed behavioral patterns that go beyond risk allocation. First, one might think that the observed behavioral changes may also result from high background risk individuals habituating more to the tactile (vibrational) cues emerging from opening and closing the lid when they received their regular background cue treatment. However, when moving tanks to the experimental shelf prior to the behavioral experiment, snails received a tactile/vibrational cue substantially larger in magnitude (and more comparable to the level of disturbance they received during their past regular background treatment that was applied alongside a water change) compared to the careful lifting and closing of the lid that we conducted during the experiment. Despite this, snail crawl-out behavior did not differ between background risk treatments during the pre-stimulus period where chemical cues were absent. This suggests that high background risk snails did not habituate to tactile cues more than low-risk ones did. Second, one may think that high background risk induced morphological defenses, which might make behavioral responses unnecessary. While on an individual level, we found evidence for the background risk treatment to induce tendentially thicker and significantly wider shells (see Supplementary Material §2), both these effects were only tendential when averaging morphology across experimental tanks (see Supplementary Material §3). In tanks receiving high-risk stimuli, we were unable to find statistical evidence for morphological defense expression to be related to crawl-out behavior (see Supplementary Material §4). Interestingly, in response to low-risk stimuli we found that individuals with greater morphological defenses (in terms of both shell thickness and shell shape) displayed *more* crawl-out behavior than less-defended conspecifics (see Supplementary Material §4). Thus, rather than supporting the hypothesis that well-defended snails do not require to respond behaviorally, our results suggest that in *P. acuta*, at least in low-risk situations, behavioral and morphological defense expression is positively correlated. However, we suggest caution in speculating further on these results, partly due to the lower sample size, and also due to the fact that we only correlated average morphological defenses across many individuals with average behavioral responses of the same groups.

In addition to our partial support for the risk allocation hypothesis, our result that irrespective of background risk, *P. acuta* increases crawl-out behavior in response towards a high-risk stimulus is in accordance with previous research (Covich et al. 1994; Tariel et al. 2020a, 2020b; Tariel-Adam et al. 2023; Meuthen and Mutingwende 2025) and other gastropods (Alexander and Covich 1991; Dalesman et al. 2007, 2009; Dalesman and Rundle 2010). This behavior is in accordance with the threat-sensitivity hypothesis, where prey animals are expected to show greater antipredator behavior during high-risk periods (Helfman 1989). Thus, this result adds to the widespread evidence for threat-sensitivity across diverse taxa (Helfman 1989; Rochette et al. 1997; Ferrari and Chivers 2010; Roux et al. 2014; Wood and Moore 2020).

Snail size, as measured in a subset of sampled snails, appears to be unaffected by our background treatments. Evidence on the impact of perceived risk on *P. acuta* size has hitherto been mixed with some studies reporting risk-induced stunted growth due to resource reallocation into defenses (Beaty et al. 2016; Meuthen and Reinhold 2023) and others reporting increased growth for the purpose of reaching a size refuge that confers safety from predators (Auld and Relyea 2011; Tariel et al. 2020b; Tariel-Adam et al. 2023). Taken together with the fact that we could not find evidence for size differences between low and high background risk snails, we speculate that size-related antipredator plasticity in *P. acuta* is specific to the evolutionary background of the studied population and specific to the predator used to simulate perceived high risk. Furthermore, our additional models that incorporated morphology (Table S2) revealed that differences in average shell sizes as well as the level of size homogeneity within groups did not impact their responses to risk. This is surprising as both larger snails (Auld and Relyea 2011) as well as prey individuals that do not stand out from the rest of the group (oddity effect, see Ohguchi 1978; Landeau and Terborgh 1986) should be subject to lower predation risk and our results also contrast the findings of previous research on fish shoals (Meuthen et al. 2016, 2021). An explanation may be that the opportunity for *P. acuta* to accurately assess the size of their group members for the purpose of involving this information in their decision-making processes is limited. This is because our study species did not evolve a visual system (eyes of pulmonate gastropods such as *Physella* sp. are pit eyes, which are mainly used for dark/light discrimination and it is unclear whether the sometimes available lenses can produce an image, see Marina and Victor Benno (2008)) or brain morphology that allows for this feat. Even though *P. acuta* can assess conspecific sizes via tactile cues (Ohbayashi-Hodoki et al. 2004), tactile communication with all surrounding snails does not arise at all times and hence it appears implausible that tactile communication can be used to accurately estimate the average size of conspecifics in close proximity during a given predation event. This theory is supported by other studies that found juvenile fish with incomplete sensory development appear to be incapable of group member size assessment as well (Meuthen et al. 2019).

Altogether, our results add to previous evidence for optimal decision-making of prey by providing support for the threat-sensitivity hypothesis and partial support for the risk allocation hypothesis under reliable long-term information about risk. They also stimulate many follow-up research avenues. First and foremost, our observation that high background risk snails may interpret tactile cues that co-occur with control water injections as sufficient evidence for high risk, may contribute to a better understanding of why evidence for risk allocation is mixed in gastropods so far. That is because tactile cues that co-occur with chemical cues are almost omnipresent during experimental settings. Hence, future studies on gastropod risk allocation should include plans to minimize the co-occurrence of tactile cues. Doing so alongside other experimental design considerations that satisfy the other conditions of the risk allocation hypothesis (see above) might make experimental evidence for gastropod risk allocation more consistent. Second, effect sizes supporting risk allocation in gastropods may be small and require larger sample sizes than the ones applied here. To investigate this possibility, the present study should be replicated with larger sample sizes. Third, we may not have sufficiently energy-limited snails (see above). Hence, future researchers could replicate our study by crossing our background treatments with different levels of food availability during development. Fourth, risk allocation could evolve only in populations or species that experienced constant levels of high risk over evolutionary history (Meuthen et al. 2019), which should stimulate replications of the present study using different *P. acuta* populations. Fifth, to further investigate the theory that crawl-out behavior may be linked to morphological defense expression in *P. acuta*, future researchers should investigate this question on an individual level, by relating variation in individual morphology to variation in individual crawl-out behavior. Lastly, due to our lack of knowledge about physiological effects of predation risk in gastropods, we can so far only speculate regarding the underlying mechanics of risk allocation. In vertebrates, it is known that perceived high risk induces fear-response related physiological changes such as altered brain dendritic morphology, neural gene expression and brain cell-cell interactions (Mitra et al. 2009; Cui et al. 2024) and altered stress hormone (glucocorticoid) production (Sheriff et al. 2011; Maher et al. 2013; Stein and Hoke 2022). As gastropods also exhibit comparable stress hormone responses that involve corticotropin (Ottaviani et al. 1992), which is a hormone that stimulates the release of glucocorticoids in vertebrates, we hypothesize that despite large physiological differences, the mechanics underlying risk allocation might be comparable to vertebrates. A particularly interesting follow-up study would thus be to study these mechanisms in gastropods by tracking stress hormone levels as well as brain gene expression in response to elevated background risk as well as to a single high-risk stimulus.

## Supplementary Material

araf078_suppl_Supplementary_Materials_1

## Data Availability

Analyses reported in this article can be reproduced using the data provided by Meuthen (2025a) and the code provided by Meuthen (2025b).

## References

[CIT0001] Adams DB , Collyer M, Kaliontzopoulou A, Baken E. 2021. geomorph: Geometric morphometric analyses of 2D/3D landmark data. R package version 4.0.0. https://doi.org/10.32614/CRAN.package.geomorph

[CIT0002] Alexander JE , Covich AP. 1991. Predator avoidance by the freshwater snail *Physella virgata* in response to the crayfish *Procambarus simulans*. Oecologia. 87:435–442. https://doi.org/10.1007/BF00634603 28313274

[CIT0003] Auld JR , Relyea RA. 2010. Life-history plasticity and inbreeding depression under mate limitation and predation risk: cumulative lifetime fitness dissected with a life table response experiment. Evol Ecol. 24:1171–1185. https://doi.org/10.1007/s10682-010-9357-6

[CIT0004] Auld JR , Relyea RA. 2011. Adaptive plasticity in predator-induced defenses in a common freshwater snail: altered selection and mode of predation due to prey phenotype. Evol Ecol. 25:189–202. https://doi.org/10.1007/s10682-010-9394-1

[CIT0005] Batabyal A , Lukowiak K. 2023. Tracking the path of predator recognition in a predator-naive population of the pond snail. Behav Ecol. 34:125–135. https://doi.org/10.1093/beheco/arac107

[CIT0006] Bates D , Mächler M, Bolker B, Walker S. 2015. Fitting linear mixed-effects models using lme4. J Stat Softw. 1:1–48. https://doi.org/10.18637/jss.v067.i01

[CIT0007] Beaty LE , et al 2016. Shaped by the past, acting in the present: transgenerational plasticity of anti-predatory traits. Oikos. 125:1570–1576. https://doi.org/10.1111/oik.03114

[CIT0008] Ben-Shachar MS , Lüdecke D, Makowski D. 2020. effectsize: estimation of effect size indices and standardized parameters. J Open Source Softw. 5:2815. https://doi.org/10.21105/joss.02815

[CIT0009] Blumstein DT , Bouskila A. 1996. Assessment and decision making in animals: a mechanistic model underlying behavioral flexibility can prevent ambiguity. Oikos. 77:569–576. https://doi.org/10.2307/3545948

[CIT0010] Boudreau ML , Scrosati RA, Wong MC. 2018. Predator (*Carcinus maenas*) nonconsumptive limitation of prey (*Nucella lapillus*) feeding depends on prey density and predator cue type. J Ethol. 36:259–264. https://doi.org/10.1007/s10164-018-0557-9

[CIT0011] Budaev S , Jørgensen C, Mangel M, Eliassen S, Giske J. 2019. Decision-making from the animal perspective: bridging ecology and subjective cognition. Front Ecol Evol. 7:164. https://doi.org/10.3389/fevo.2019.00164

[CIT0012] Colegrave N , Ruxton GD. 2017. Statistical model specification and power: recommendations on the use of test-qualified pooling in analysis of experimental data. Proc Roy Soc B. 284:20161850. https://doi.org/10.1098/rspb.2016.1850 PMC5378071 28330912

[CIT0013] Colley DG , Bustinduy AL, Secor WE, King CH. 2014. Human schistosomiasis. Lancet (London, England). 383:2253–2264. https://doi.org/10.1016/S0140-6736(13)61949-2 24698483 PMC4672382

[CIT0014] Conner SL , Pomory CM, Darby PC. 2008. Density effects of native and exotic snails on growth in juvenile apple snails *Pomacea paludosa* (Gastropoda: Ampullariidae): a laboratory experiment. J Molluscan Stud. 74:355–362. https://doi.org/10.1093/mollus/eyn024

[CIT0015] Covich AP , Crowl TA, Alexander JE, Vaughn CC. 1994. Predator-avoidance responses in freshwater decapod-gastropod interactions mediated by chemical stimuli. J North Am Benthol Soc. 13:283–290. https://doi.org/10.2307/1467246

[CIT0016] Cui K , et al 2024. Dominant activities of fear engram cells in the dorsal dentate gyrus underlie fear generalization in mice. PLoS Biol. 22:e3002679. https://doi.org/10.1371/journal.pbio.3002679 38995985 PMC11244812

[CIT0017] Dalesman S , Rundle SD. 2010. Influence of rearing and experimental temperatures on predator avoidance behaviour in a freshwater pulmonate snail. Freshw Biol. 55:2107–2113. https://doi.org/10.1111/j.1365-2427.2010.02470.x

[CIT0018] Dalesman S , Rundle SD, Cotton PA. 2007. Predator regime influences innate anti-predator behaviour in the freshwater gastropod *Lymnaea stagnalis*. Freshw Biol. 52:2134–2140. https://doi.org/10.1111/j.1365-2427.2007.01843.x

[CIT0019] Dalesman S , Rundle SD, Cotton PA. 2009. Crawl-out behaviour in response to predation cues in an aquatic gastropod: insights from artificial selection. Evol Ecol. 23:907–918. https://doi.org/10.1007/s10682-008-9280-2

[CIT0020] Dan NA , Bailey SER. 1982. Growth, mortality, and feeding rates of the snail *Helix aspersa* at different population densities in the laboratory, and the depression of activity of helicid snails by other individuals, or their mucus. J Molluscan Stud. 48:257–265. https://doi.org/10.1093/oxfordjournals.mollus.a065647

[CIT0021] DeWitt TJ , Prestridge HL. 2022. On the need for antibiotics to reduce subject losses and biases in experiments with aquatic molluscs. Malacologia. 64:303–307. https://doi.org/10.4002/040.064.0211

[CIT0022] DeWitt TJ , Sih A, Hucko JA. 1999. Trait compensation and cospecialization in a freshwater snail: size, shape and antipredator behaviour. Anim Behav. 58:397–407. https://doi.org/10.1006/anbe.1999.1158 10458891

[CIT0023] Donelan SC , Trussell GC. 2018. Parental and embryonic experiences with predation risk affect prey offspring behaviour and performance. Proc Biol Sci. 285:20180034. https://doi.org/10.1098/rspb.2018.0034 29540520 PMC5879633

[CIT0087] Draparnaud J. 1805. Histoire naturelle des mollusques terrestres et fluviatiles de la France. Paris: Chez J.-B. Bailliere.

[CIT0024] Engqvist L. 2005. The mistreatment of covariate interaction terms in linear model analyses of behavioural and evolutionary ecology studies. Anim Behav. 70:967–971. https://doi.org/10.1016/j.anbehav.2005.01.016

[CIT0025] Ferrari MCO , Chivers DP. 2010. The ghost of predation future: threat-sensitive and temporal assessment of risk by embryonic woodfrogs. Behav Ecol Sociobiol. 64:549–555. https://doi.org/10.1007/s00265-009-0870-y

[CIT0026] Ferrari MCO , Sih A, Chivers DP. 2009. The paradox of risk allocation: a review and prospectus. Anim Behav. 78:579–585. https://doi.org/10.1016/j.anbehav.2009.05.034

[CIT0027] FitzGibbon CD. 1994. The costs and benefits of predator inspection behaviour in Thomson’s gazelles. Behav Ecol Sociobiol. 34:139–148. https://doi.org/10.1007/bf00164184

[CIT0028] Goeppner SR , Roberts ME, Beaty LE, Luttbeg B. 2020. Freshwater snail responses to fish predation integrate phenotypic plasticity and local adaptation. Aquat Ecol. 54:309–322. https://doi.org/10.1007/s10452-019-09744-x

[CIT0029] Hamilton IM , Heithaus MR. 2001. The effects of temporal variation in predation risk on anti-predator behaviour: an empirical test using marine snails. Proc Biol Sci. 268:2585–2588. https://doi.org/10.1098/rspb.2001.1857 11749714 PMC1088919

[CIT0030] Helfman GS. 1989. Threat-sensitive predator avoidance in damselfish-trumpetfish interactions. Behav Ecol Sociobiol. 24:47–58. https://doi.org/10.1007/bf00300117

[CIT0031] Hoverman JT. 2010. Predator-induced plasticity. Encyclopedia of Life Sciences (ELS) Chichester: John Wiley & Sons.

[CIT0032] Kain MP , McCoy MW. 2016. Anti-predator behavioral variation among *Physa acuta* in response to temporally fluctuating predation risk by *Procambarus*. Behav Process. 133:15–23. https://doi.org/10.1016/j.beproc.2016.10.011 27984081

[CIT0033] Lakowitz T , Brönmark C, Nyström P. 2008. Tuning in to multiple predators: conflicting demands for shell morphology in a freshwater snail. Freshw Biol. 53:2184–2191. https://doi.org/10.1111/j.1365-2427.2008.02045.x

[CIT0034] Landeau L , Terborgh J. 1986. Oddity and the confusion effect in predation. Anim Behav. 34:1372–1380. https://doi.org/10.1016/s0003-3472(86)80208-1

[CIT0035] Lawrence BJ , Smith RJF. 1989. Behavioral-response of solitary fathead minnows, *Pimephales promelas*, to alarm substance. J Chem Ecol. 15:209–219. https://doi.org/10.1007/BF02027783 24271436

[CIT0036] Lenth RV , Buerkner P, Herve M, Love J, Riebl H, Singmann H. 2020. Emmeans: estimated marginal means, aka least-squares means. R package version 1.10.3. https://doi.org/10.32614/CRAN.package.emmeans

[CIT0037] Lima SL. 1998. Stress and decision making under the risk of predation: recent developments from behavioral, reproductive, and ecological perspectives. Adv Stud Behav. 27:215–290. https://doi.org/10.1016/S0065-3454(08)60366-6

[CIT0038] Lima SL , Bednekoff PA. 1999. Temporal variation in danger drives antipredator behavior: the predation risk allocation hypothesis. Am Nat. 153:649–659. https://doi.org/10.1086/303202 29585647

[CIT0039] Lima SL , Dill LM. 1990. Behavioral decisions made under the risk of predation - a review and prospectus. Can J Zool. 68:619–640. https://doi.org/10.1139/z90-092

[CIT0040] Lüdecke D , Bartel A, Schwemmer C, Powell C, Djalovski A, Titz J. 2024a. sjPlot: data visualization for statistics in social science. R package version 2.8.16. https://doi.org/10.32614/CRAN.package.sjPlot

[CIT0041] Lüdecke D , et al 2024b. performance: assessment of regression models performance. R package version 0.12.2. https://doi.org/10.32614/CRAN.package.performance

[CIT0042] Luttbeg B. 2017. Re-examining the causes and meaning of the risk allocation hypothesis. Am Nat. 189:644–656. https://doi.org/10.1086/691470 28514637

[CIT0043] Lyon P. 2015. The cognitive cell: bacterial behavior reconsidered. Front Microbiol. 6:264. https://doi.org/10.3389/fmicb.2015.00264 25926819 PMC4396460

[CIT0044] Maher JM , Werner EE, Denver RJ. 2013. Stress hormones mediate predator-induced phenotypic plasticity in amphibian tadpoles. Proc Roy Soc B. 280:20123075. https://doi.org/10.1098/rspb.2012.3075 PMC3619459 23466985

[CIT0045] Marina VZ , Victor Benno M-R. 2008. Understanding the cephalic eyes of pulmonate gastropods: a review. Am Malacol Bull. 26:47–66. https://doi.org/10.4003/006.026.0206

[CIT0046] Mas‐Coma S , Valero MA, Bargues MD. 2009. Chapter 2 *Fasciola*, lymnaeids and human fascioliasis, with a global overview on disease transmission, epidemiology, evolutionary genetics, molecular epidemiology and control. Adv Parasitol. 69:41–146. https://doi.org/10.1016/S0065-308X(09)69002-3 19622408

[CIT0047] Matassa CM , Trussell GC. 2014. Prey state shapes the effects of temporal variation in predation risk. Proc Biol Sci. 281:20141952. https://doi.org/10.1098/rspb.2014.1952 25339716 PMC4213655

[CIT0048] McNamara JM , Houston AI. 1986. The common currency for behavioral decisions. Am Nat. 127:358–378. https://doi.org/10.1086/284489

[CIT0049] Meuthen D. 2025a. Risk allocation in a freshwater gastropod. Behav Ecol. https://doi.org/10.5061/dryad.tqjq2bwbb PMC12343016 40799770

[CIT0050] Meuthen D. 2025b. Code from: Risk allocation in a freshwater gastropod. Zenodo. https://doi.org/10.5281/zenodo.15781562 PMC12343016 40799770

[CIT0051] Meuthen D , Baldauf SA, Bakker TCM, Thünken T. 2016. Predator-induced neophobia in juvenile cichlids. Oecologia. 181:947–958. https://doi.org/10.1007/s00442-015-3478-0 26578223

[CIT0052] Meuthen D , Ferrari MCO, Chivers DP. 2021. Paternal care effects outweigh gamete-mediated and personal environment effects during the transgenerational estimation of risk in fathead minnows. BMC Ecol Evol. 21:187. https://doi.org/10.1186/s12862-021-01919-1 34635051 PMC8507329

[CIT0053] Meuthen D , Ferrari MCO, Lane T, Chivers DP. 2019. High background risk induces risk allocation rather than generalized neophobia in the fathead minnow. Behav Ecol. 30:1416–1424. https://doi.org/10.1093/beheco/arz094

[CIT0054] Meuthen D , Mutingwende N. 2025. Chilled but potent: validating the ability of frozen alarm cues to induce antipredator defences in a freshwater gastropod. Anim Behav 226:123252. https://doi.org/10.1016/j.anbehav.2025.123252

[CIT0055] Meuthen D , Reinhold K. 2023. On the use of antibiotics in plasticity research: gastropod shells unveil a tale of caution. J Anim Ecol. 92:1055–1064. https://doi.org/10.1111/1365-2656.13909 36869422

[CIT0056] Mitra R , Adamec R, Sapolsky R. 2009. Resilience against predator stress and dendritic morphology of amygdala neurons. Behav Brain Res. 205:535–543. https://doi.org/10.1016/j.bbr.2009.08.014 19686780 PMC4022315

[CIT0057] Morris JP , Backeljau T, Chapelle G. 2019. Shells from aquaculture: a valuable biomaterial, not a nuisance waste product. Rev Aquacult. 11:42–57. https://doi.org/10.1111/raq.12225

[CIT0058] Ohbayashi-Hodoki K , Ishihama F, Shimada M. 2004. Body size–dependent gender role in a simultaneous hermaphrodite freshwater snail, *Physa acuta*. Behav Ecol. 15:976–981. https://doi.org/10.1093/beheco/arh101

[CIT0059] Ohguchi O. 1978. Experiments on selection against color oddity of water fleas by three-spined sticklebacks. Z Tierpsychol. 47:254–267. https://doi.org/10.1111/j.1439-0310.1978.tb01835.x

[CIT0060] Osborne TR , Stehman SV. 2022. Improving external shell volume estimation in snails using landmark-based size measurements. J Molluscan Stud. 88:eyac032. https://doi.org/10.1093/mollus/eyac032

[CIT0061] Ottaviani E , Caselgrandi E, Petraglia F, Franceschi C. 1992. Stress response in the freshwater snail *Planorbarius corneus* (L.) (Gastropoda, Pulmonata): interaction between CRF, ACTH, and biogenic amines. Gen Comp Endocrinol. 87:354–360. https://doi.org/10.1016/0016-6480(92)90041-h 1330806

[CIT0062] R Core Team. 2024. R: A language and environment for statistical computing. Vienna, Austria: R Foundation for Statistical Computing.

[CIT0063] Rochette R , Dill LM, Himmelman JH. 1997. A field test of threat sensitivity in a marine gastropod. Anim Behav. 54:1053–1062. https://doi.org/10.1006/anbe.1997.0488 9398362

[CIT0064] Rohlf FJ. 2015. The tps series of software. Hystrix. 26:9–12. https://doi.org/10.4404/hystrix-26.1-11264

[CIT0065] Roux O , Diabaté A, Simard F. 2014. Divergence in threat sensitivity among aquatic larvae of cryptic mosquito species. J Anim Ecol. 83:702–711. https://doi.org/10.1111/1365-2656.12163 24138173

[CIT0066] Salice CJ , Plautz SC. 2011. Predator-induced defences in offspring of laboratory and wild-caught snails: prey history impacts prey response. Evol Ecol Res. 13:373–386. https://www.evolutionary-ecology.com/abstracts/v13/2664.html

[CIT0067] Sheriff MJ , Krebs CJ, Boonstra R. 2011. From process to pattern: how fluctuating predation risk impacts the stress axis of snowshoe hares during the 10-year cycle. Oecologia. 166:593–605. https://doi.org/10.1007/s00442-011-1907-2 21246218

[CIT0069] Sih A. 1992. Prey uncertainty and the balancing of antipredator and feeding needs. Am Nat. 139:1052–1069. https://doi.org/10.1086/285372

[CIT0070] Sih A , McCarthy TM. 2002. Prey responses to pulses of risk and safety: testing the risk allocation hypothesis. Anim Behav. 63:437–443. https://doi.org/10.1006/anbe.2001.1921

[CIT0071] Sih A , Ziemba R, Harding KC. 2000. New insights on how temporal variation in predation risk shapes prey behavior. Trends Ecol Evol. 15:3–4. https://doi.org/10.1016/s0169-5347(99)01766-8 10603494

[CIT0072] Slos S , Meester LD, Stoks R. 2009. Behavioural activity levels and expression of stress proteins under predation risk in two damselfly species. Ecol Entomol. 34:297–303. https://doi.org/10.1111/j.1365-2311.2008.01077.x

[CIT0073] Stein LR , Hoke K. 2022. Parental and individual experience with predation risk interact in shaping phenotypes in a sex-specific manner. Anim Behav. 191:75–89. https://doi.org/10.1016/j.anbehav.2022.06.012

[CIT0074] Suddendorf T , Bulley A, Miloyan B. 2018. Prospection and natural selection. Curr Opin Behav Sci. 24:26–31. https://doi.org/10.1016/j.cobeha.2018.01.019

[CIT0076] Tariel J , Plénet S, Luquet E. 2020a. How do developmental and parental exposures to predation affect personality and immediate behavioural plasticity in the snail *Physa acuta*? Proc Biol Sci. 287:20201761. https://doi.org/10.1098/rspb.2020.1761 33352075 PMC7779491

[CIT0077] Tariel J , Plénet S, Luquet E. 2020b. Transgenerational plasticity of inducible defenses: combined effects of grand-parental, parental and current environments. Ecol Evol. 10:2367–2376. https://doi.org/10.1002/ece3.6046 32184987 PMC7069331

[CIT0075] Tariel-Adam J , Luquet E, Plénet S. 2023. Sensitive windows for within- and trans-generational plasticity of anti-predator defences. Peer Community J. 3:e71. https://doi.org/10.24072/pcjournal.304

[CIT0078] Teplitsky C , Plenet S, Joly P. 2005. Costs and limits of dosage response to predation risk: to what extent can tadpoles invest in anti-predator morphology? Oecologia. 145:364–370. https://doi.org/10.1007/s00442-005-0132-2 16001226

[CIT0079] Trussell GC , Matassa CM, Luttbeg B. 2011. The effects of variable predation risk on foraging and growth: Less risk is not necessarily better. Ecology. 92:1799–1806. https://doi.org/10.1890/10-2222.1 21939076

[CIT0080] Turner AM. 1996. Freshwater snails alter habitat use in response to predation. Anim Behav. 51:747–756. https://doi.org/10.1006/anbe.1996.0079

[CIT0081] Turner AM , Montgomery SL. 2003. Spatial and temporal scales of predator avoidance: Experiments with fish and snails. Ecology. 84:616–622. https://doi.org/10.1890/0012-9658(2003)084[0616:satsop]2.0.co;2

[CIT0082] Vinarski MV. 2017. The history of an invasion: phases of the explosive spread of the physid snail *Physella acuta* through Europe, Transcaucasia and Central Asia. Biol Invasions. 19:1299–1314. https://doi.org/10.1007/s10530-016-1339-3

[CIT0083] Wijsman JWM , Troost K, Fang J, Roncarati A. 2019. Global production of marine bivalves. Trends and challenges. In: Smaal AC, Ferreira JG, Grant J, Petersen JK, Strand Ø, editors. Goods and services of marine bivalves. Cham: Springer International Publishing. p. 7–26.

[CIT0084] Wisenden BD. 2015. Chemical cues that indicate risk of predation. In: Sorensen C, Wisenden BD, editors. Fish pheromones and related cues. Oxford: Wiley Blackwell. p. 131–148.

[CIT0085] Wood TC , Moore PA. 2020. Fine-tuned responses to chemical landscapes: crayfish use predator odors to assess threats based on relative size ratios. Ecosphere. 11:e03188. https://doi.org/10.1002/ecs2.3188

[CIT0086] Zelditch ML , Swiderski DL, Sheets HD, editors. 2012. Geometric morphometrics for biologists: a primer, 2nd ed. London: Academic Press.

