# Supplementary material for: Risk allocation in a freshwater gastropod
Source: Behav Ecol. 2025 Jul 13;36(4):araf078. doi: 10.1093/beheco/araf078 (PMC12343016; doi:10.1093/beheco/araf078)
Supplement: araf078_suppl_Supplementary_Materials_1 [file araf078_suppl_supplementary_materials_1.docx]

**Risk allocation in a freshwater gastropod**

Denis Meuthen

**Supplementary Information**

**§ 1 Effects of background risk treatments on mortality**

We also investigated whether the proportion of mortality, average size and snail homogeneity differed between background risk treatments. To this end, we constructed (generalized) linear mixed effect models where we sequentially entered one of these factors as dependent variable, background risk (low-risk/high-risk) as a fixed effect and family as a random intercept. We did not enter tank identity as a random factor here as every tank only provided a single datapoint here. In gaussian models, we confirmed the normality of residuals using Kolmogorov-Smirnov tests with Lilliefors correction and the homogeneity of variances using Levene tests. We ascertained that none of our binomial models was overdispersed with the performance R package v. 0.12.2 (Lüdecke et al. 2024); in gaussian models, we instead confirmed the normality of residuals using Kolmogorov-Smirnov tests with Lilliefors correction and the homogeneity of variances using Levene tests. Statistical inferences were drawn the same way as described in the main manuscript.

Over the raising period, 11 offspring died (dead snails per tank, median, [quartiles], range: 0, [0-0], 0-2) but we did not find evidence for the the proportion of dead snails to differ statistically between background risk treatments as both the high-risk treatment (0.042±0.083) and the low-risk treatment (0.035±0.105) had comparable mortality (estimated difference: -0.283 [-2.037, 1.001], d_Cohen_=0.119 [-0.371, 0.609], Χ²=0.228, p=0.633).

**§ 2 Effects of background risk treatments on individual snail morphology**

To assess individual morphology, after the crawl-out trials concluded, we blotted individual snails dry with paper towels, placed it on a ~1mm² piece of play dough to ensure that their aperture opening was parallel to the camera on all axes and photographed them, aperture upwards, on top of a size standard, at 2048 x 1536 pixel resolution with a Moticam 1080 attached to a SMZ-171 stereomicroscope (Motic, Xiamen, China). Immediately afterwards, we used a digital caliper (Model ABS-AOS 500-181-30, Mitutoyo, Kawasaki, Japan) to measure individual shell thickness to the nearest 0.01 mm three times: at the top, at the centre and at the bottom of the aperture. These three measurents per individual were then used to calculate an average value as done in in previous studies (Meuthen and Reinhold 2023).

All images were evaluated using geometric morphometrics (Zelditch et al. 2012). With tpsDig2 v.2.30 (Rohlf 2015) after size calibration, we placed 11 established (Beaty et al. 2016) landmarks along with 21 semilandmarks on each photograph. Using the geomorph R package v.4.0.7 (Adams et al. 2021), we first performed a Procrustes superimposition followed by a principal component analysis. In addition, we extracted shell centroid size (i.e., the square root of the sum of distances squared from each landmark to the centroid), a robust proxy for shell volume in gastropods (Osborne and Stehman 2022).

By applying Cattell’s Scree test (Morton and Altschul 2019), we extracted five principal components that explained in total 80.06% of shape variation (PC1: 37.33%, PC2:17.22%, PC3:15.00%, PC4:5.40%, PC5: 5.11%). The first and second principal components loaded mainly on aperture size, the third one on a combination of aperture size and shell width (separating short, rotund shells with large apertures from elongated shells with small apertures), and both the fourth and fifth one loaded only negligibly on the same trait combination. Thus, only the first three components were strongly related to known antipredator defenses in *P. acuta* (i.e., wider shells, smaller apertures), and were selected for downstream analyses.

We first aimed to determine whether individuals from different background risk treatments differed in their morphology. To this end, we constructed linear mixed-effect models (LMEs) with maximum likelihood estimation using the LME4 R package v.1.1-35.3 (Bates et al. 2015). Here, one-by-one we entered the respective trait (centroid size, PC1-3 shell shape, and average shell thickness as dependent variable). Background treatment (high-risk/low-risk) was then entered as fixed effect and family identity nested in tank identity as random intercept. We investigated these models in the same way as described in the main manuscript.

Regarding shell centroid size, we did not find evidence for high background risk individuals to differ from low background risk ones (estimated difference: 0.056 [-0.418, 0.53] mm², d_Cohen_=-0.1 [-0.951, 0.753], Χ²=0.053, p=0.817). Shells tended to be thicker in high-risk snails compared to low-risk individuals (estimated difference: -0.006 [-0.014, 0.001], d_Cohen_=0.705 [-0.142, 1.537], Χ²=2.807, p=0.094). Furthermore, we did not find statistical evidence for a difference in body shape between high-risk and low-risk snails among the first (estimated difference: 0.003 [-0.004, 0.009], d_Cohen_=-0.347 [-1.2, 0.515], Χ²=0.639, p=0.424) and second principal component (estimated difference: -0.004 [-0.01, 0.002], d_Cohen_=0.516 [-0.315, 1.336], Χ²=1.567, p=0.211), which both loaded on variation in aperture size. The third principal component (PC3), which loaded on both shell width and aperture size, however, differed significantly between high-risk and low-risk snails (estimated difference: 0.005 [0.001, 0.009], d_Cohen_=-1.077 [-1.976, -0.156], Χ²=5.95, p=0.015). High-risk snails developed short, rotund shells with large apertures as opposed to low-risk snails which had elongated shells with small apertures.

**§ 3 Effects of background risk treatments on tank-wise average snail morphology**

To allow for comparison with behavioral data, for each tank, we then calculated average snail size (i.e., mean centroid size) as well as coefficients of variation as proxy for snail homogeneity within tanks (CV, i.e., dividing the standard deviation by the mean). In addition, for each tank, we averaged shell thicknesses, as well as PC3 scores. We did not find statistical evidence for our background treatment to significantly affect average snail size (estimated difference: 0.073 [-0.234, 0.379], d_Cohen_=-0.197 [-1.014, 0.623], Χ²=0.233, p=0.629), snail homogeneity (estimated difference: -0.001 [-0.025, 0.022], d_Cohen_=0.052 [-0.765, 0.867], Χ²=0.016, p=0.899), average shell thickness (estimated difference: -0.011 [-0.023, 0.001], d_Cohen_=0.753 [-0.097, 1.588], Χ²=3.185, p=0.074) or average shell shape within tanks (estimated difference: 0.004 [0, 0.008], d_Cohen_=-0.775 [-1.613, 0.079], Χ²=3.367, p=0.067).

**§ 4 Effects of tank-wise average snail morphology on crawl-out behavior**

First, we assessed whether including average snail size and snail homogeneity (i.e., CV of size) in our models alters our main findings regarding snail behaviour. To this end, using a reduced dataset (i.e., a dataset containing only the tanks of which we had size-related information), we created the same model aiming to study variation in crawl-out behavior as in the main manuscript but additionally included average snail size and snail homogeneity as fixed effects. We did not include any interactions these new covariates and the other fixed effects as our number of samples was too low to obtain reliable estimates for such interactions. We investigated these models in the same way as described in the main manuscript. Results are shown in Table S2.

Second, we assessed whether the response towards experimental stimuli was driven by the extent of developed morphological defenses rather than background risk. To this end, we constructed two models where, using the same model specification outlined in the main manuscript, we replaced the fixed effect background treatment with average shell thickness or average shell shape, respectively. Based on the observation that the interaction with interval was hitherto always non-significant, we now excluded it from the fixed effect structure while retaining the same random effect structure as before. Here, we first found statistical evidence for the presence of a three-way interaction involving average shell thickness (interaction average shell thickness × stimulus treatment × phase, LRT, Χ²=8.141, p=0.043). When we disentangled this interaction, in tanks that received high-risk stimuli, we found no statistical evidence for shell thickness to be correlated with either prestimulus (estimated slope: -2.670 [-16.62, 11.30], z=-0.376, p=0.707) or post-stimulus crawl-out behavior (estimated slope: 3.290 [-11.348, 17.900], z=0.440, p=0.660). However, in tanks that received low-risk stimuli, we found statistical evidence for average shell thickness to be correlated with the crawl-out response tendentially during the prestimulus (estimated slope: 19.340 [-0.544, 39.200], z=1.906, p=0.057) and significantly during the post-stimulus period (estimated slope: 32.030 [11.553, 52.500], z=3.066, p=0.002). This means that snails that had on average thicker shells crawled out *more* after receiving a low-risk stimulus than tanks containing thin-shelled conspecifics, suggesting that morphological and behavioral antipredator defenses may be correlated. In terms of shell shape, we observed only a tendential interactive effect (interaction average shell shape × stimulus treatment × phase, LRT, Χ²=6.931, p=0.074). However, when disentangling this interaction, we found similar effects as for shell thickness. In tanks that received high-risk stimuli, we found no statistical evidence for shape to be correlated with either prestimulus (estimated slope: -25.060 [-55.700, 5.620], z=-1.601, p=0.110) or post-stimulus crawl-out behavior (estimated slope: -8.290 [-36.800, 20.230], z=-0.569, p=0.569). However, in tanks that received low-risk stimuli, we found statistical evidence for average shell shape to be significantly correlated with both the crawl-out response during the prestimulus (estimated slope: -39.300 [-70.000, -8.640], z=-2.512, p=0.012) and post-stimulus period (estimated slope: -48.740 [-79.700, -17.760], z=-3.084, p=0.002). As *lower* PC3 shape scores are associated with greater morphological defenses (short and wide shells), the observed results again suggest that snails with greater morphological defenses display *more* crawl-out behavior both before and after receiving low-risk stimuli.

**Table S1** Full and final generalized linear mixed-effect models analyzing variation in the stimulus-induced change of *Physella acuta* crawl-out behavior (n_observations_=768, n_tanks_=64, n_families_=22). Statistically significant effects (p<0.05) are highlighted in bold font.

| *predictors* | *odds ratios* | *95% CI* | *p* |
| --- | --- | --- | --- |
| ***full model*** |  |  |  |
| (Intercept) | 0.18 | 0.08 – 0.39 | **<0.001** |
| background treatment | 0.74 | 0.26 – 2.13 | 0.578 |
| stimulus treatment | 0.31 | 0.10 – 0.97 | **0.045** |
| phase | 0.37 | 0.15 – 0.90 | **0.029** |
| interval within | 0.97 | 0.93 – 1.00 | 0.069 |
| background treatment × stimulus treatment | 2.78 | 0.55 – 14.03 | 0.217 |
| background treatment × phase | 0.6 | 0.15 – 2.35 | 0.462 |
| stimulus treatment × phase | 4.76 | 1.33 – 17.05 | **0.017** |
| background treatment × interval | 1.04 | 0.99 – 1.09 | 0.103 |
| stimulus treatment × interval | 1.05 | 1.00 – 1.10 | 0.058 |
| phase × interval | 1.05 | 1.00 – 1.10 | **0.042** |
| background treatment × stimulus treatment × phase | 0.5 | 0.07 – 3.32 | 0.471 |
| background treatment × stimulus treatment × interval | 0.94 | 0.87 – 1.00 | 0.062 |
| background treatment × phase × interval | 0.98 | 0.91 – 1.05 | 0.518 |
| stimulus treatment × phase × interval | 0.93 | 0.87 – 1.00 | **0.037** |
| background treatment × stimulus treatment × phase × interval | 1.07 | 0.97 – 1.17 | 0.196 |
| *random effects* | | | |
| σ^2^ | 0.56 | | |
| τ_00_ _tank:family_ | 0.71 | | |
| τ_00_ _family_ | 0.36 | | |
| τ_11_ _tank:family.interval_ | 0 | | |
| τ_11_ _family.interval_ | 0 | | |
| ρ_01_ _tank:family_ | -0.53 | | |
| ρ_01_ _family_ | -1 | | |
|  |  |  |  |
| ***final model*** |  |  |  |
| (Intercept) | 0.1 | 0.06 – 0.18 | **<0.001** |
| background treatment | 1.37 | 0.87 – 2.16 | 0.176 |
| stimulus treatment | 0.68 | 0.43 – 1.08 | 0.1 |
| phase | 0.77 | 0.55 – 1.10 | 0.148 |
| interval | 1 | 0.99 – 1.02 | 0.84 |
| background treatment × phase | 0.51 | 0.34 – 0.76 | **0.001** |
| stimulus treatment × phase | 1.7 | 1.13 – 2.55 | **0.011** |
| *random effects* | | | |
| σ^2^ | 0.56 | | |
| τ_00_ _tank:family_ | 0.7 | | |
| τ_00_ _family_ | 0.34 | | |
| τ_11_ _tank:family.interval_ | 0 | | |
| τ_11_ _family.interval_ | 0 | | |
| ρ_01_ _tank:family_ | -0.52 | | |
| ρ_01_ _family_ | -1 | | |

**Table S2** Full and final generalized linear mixed-effect models analyzing variation in the stimulus-induced change of *Physella acuta* crawl-out behavior in a reduced dataset that includes morphological information (n_observations_=504, n_tanks_=42, n_families_=20). Statistically significant effects (p<0.05) are highlighted in bold font.

| *predictors* | *odds ratios* | *95% CI* | *p* |
| --- | --- | --- | --- |
| ***full model*** |  |  |  |
| (Intercept) | 0.39 | 0.00 – 94.81 | 0.739 |
| average snail size | 0.98 | 0.74 – 1.28 | 0.867 |
| snail homogeneity | 0.04 | 0.00 – 31.62 | 0.339 |
| background treatment | 0.92 | 0.25 – 3.45 | 0.903 |
| stimulus treatment | 0.36 | 0.09 – 1.43 | 0.147 |
| phase | 0.32 | 0.11 – 0.93 | **0.035** |
| interval within | 0.97 | 0.94 – 1.01 | 0.194 |
| background treatment × stimulus treatment | 1.94 | 0.26 – 14.51 | 0.517 |
| background treatment × phase | 0.66 | 0.14 – 3.25 | 0.613 |
| stimulus treatment × phase | 6.27 | 1.38 – 28.47 | **0.017** |
| background treatment × interval | 1.02 | 0.96 – 1.07 | 0.547 |
| stimulus treatment × interval | 1.04 | 0.98 – 1.11 | 0.163 |
| phase × interval | 1.05 | 1.00 – 1.11 | 0.066 |
| background treatment × stimulus treatment × phase | 0.36 | 0.04 – 3.17 | 0.354 |
| background treatment × stimulus treatment × interval | 0.95 | 0.88 – 1.03 | 0.254 |
| background treatment × phase × interval | 0.99 | 0.91 – 1.07 | 0.77 |
| stimulus treatment × phase × interval | 0.92 | 0.85 – 1.00 | **0.038** |
| background treatment × stimulus treatment × phase × interval | 1.08 | 0.96 – 1.20 | 0.187 |
| *random effects* | | | |
| σ^2^ | 0.56 | | |
| τ_00_ _tank:family_ | 0.69 | | |
| τ_00_ _family_ | 0.3 | | |
| τ_11_ _tank:family.interval_ | 0 | | |
| τ_11_ _family.interval_ | 0 | | |
| ρ_01_ _tank:family_ | -0.82 | | |
| ρ_01_ _family_ | -0.9 | | |
|  |  |  |  |
| ***final model*** |  |  |  |
| (Intercept) | 0.26 | 0.00 – 51.69 | 0.617 |
| average snail size | 0.98 | 0.75 – 1.28 | 0.87 |
| snail homogeneity | 0.04 | 0.00 – 22.97 | 0.317 |
| background treatment | 1.16 | 0.72 – 1.87 | 0.531 |
| stimulus treatment | 0.68 | 0.42 – 1.11 | 0.122 |
| phase | 0.74 | 0.49 – 1.10 | 0.133 |
| interval | 1 | 0.98 – 1.02 | 0.958 |
| background treatment × phase | 0.62 | 0.39 – 1.00 | **0.049** |
| stimulus treatment × phase | 1.69 | 1.05 – 2.71 | **0.029** |
| *random effects* | | | |
| σ^2^ | 0.56 | | |
| τ_00_ _tank:family_ | 0.65 | | |
| τ_00_ _family_ | 0.29 | | |
| τ_11_ _tank:family.interval_ | 0 | | |
| τ_11_ _family.interval_ | 0 | | |
| ρ_01_ _tank:family_ | -0.8 | | |
| ρ_01_ _family_ | -0.95 | | |


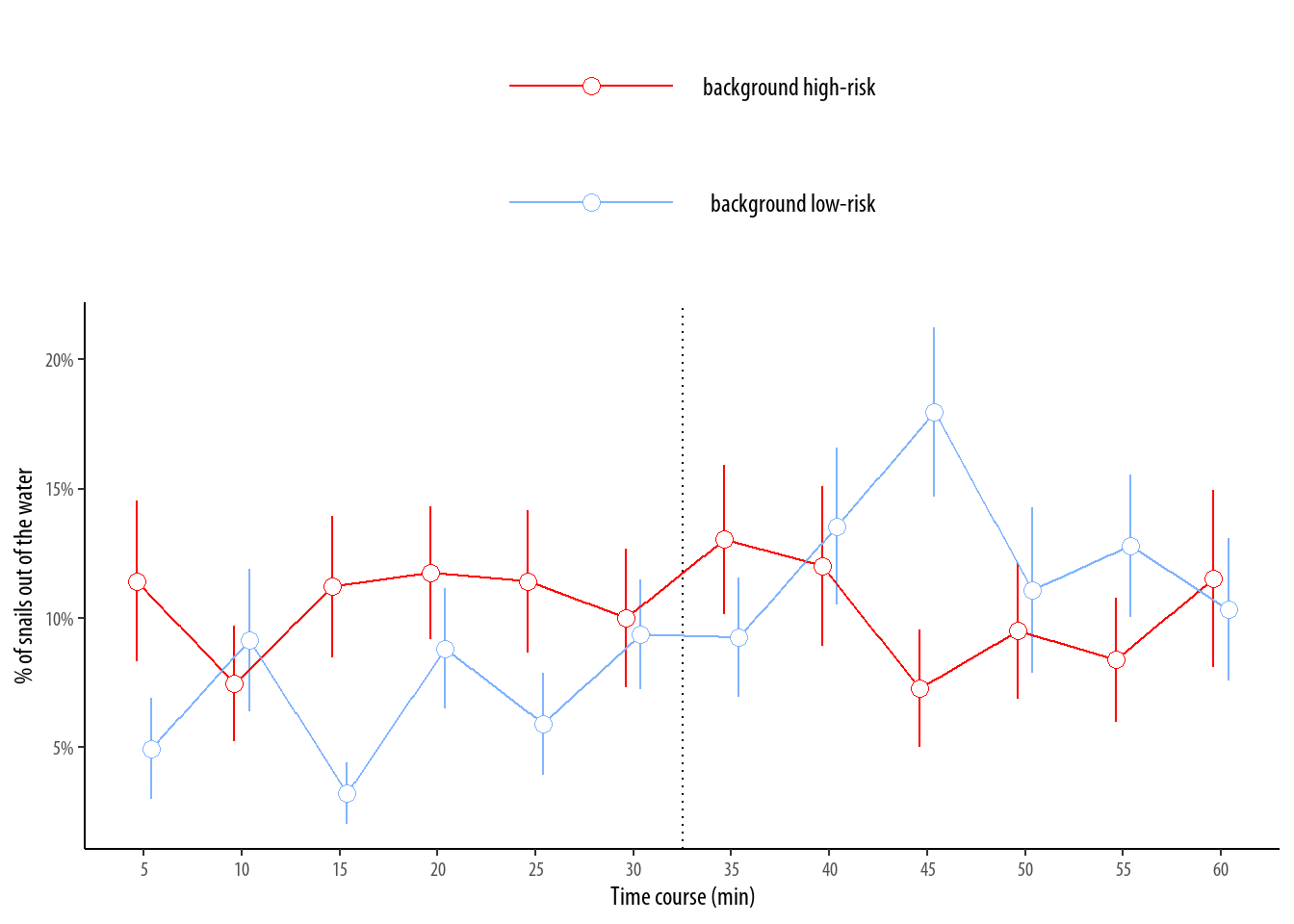


**Figure S1** Crawl-out behavior (means ± SE) as assessed in 5-minute intervals over the 60-minute experimental period for individuals that were from birth onwards continuously exposed to either high (conspecific alarm cues, red dots and lines) or low background risk (water control, blue dots and lines). The dotted line represents the timepoint at which the chemical stimulus was added to the tank.


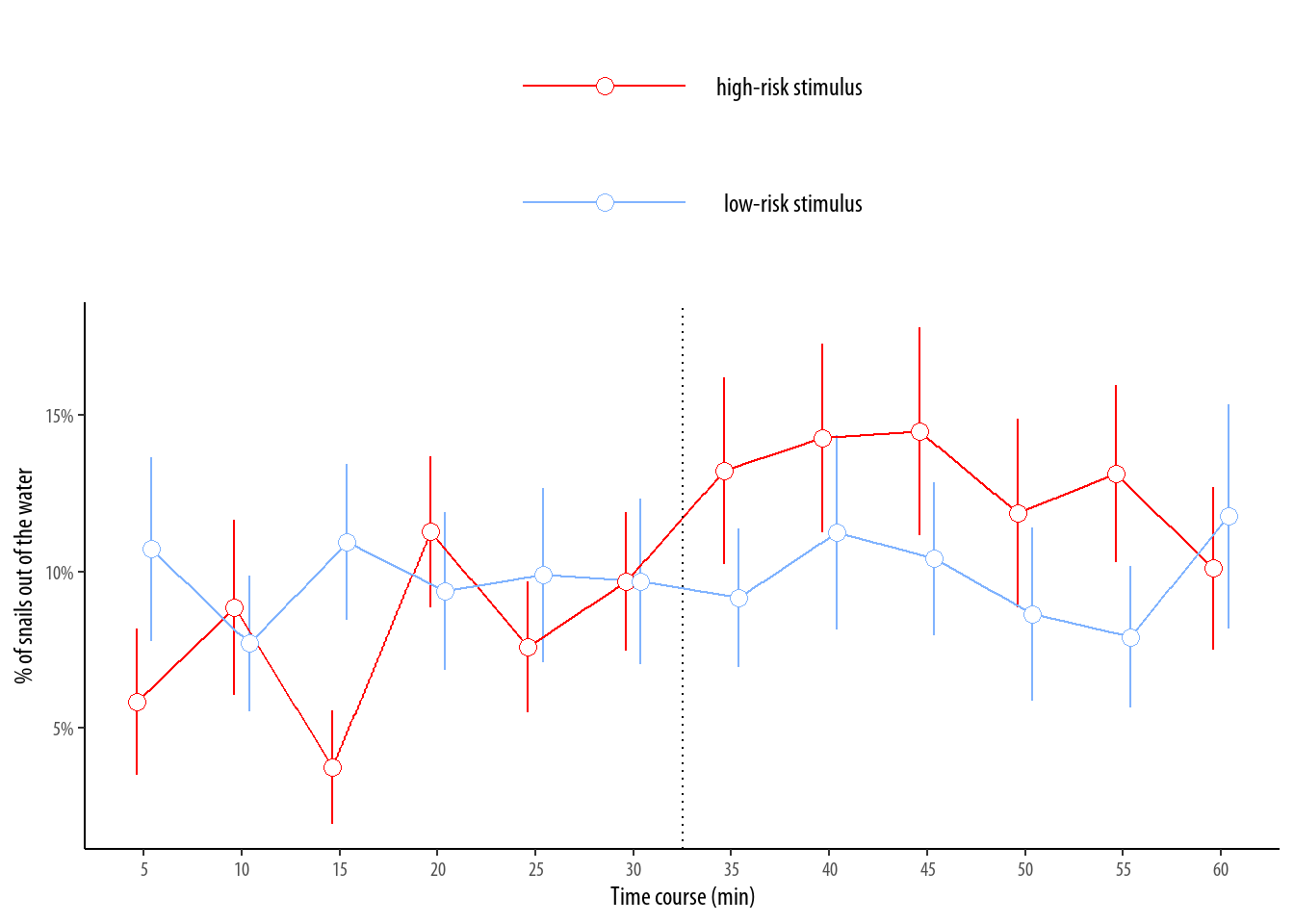


**Figure S2** Crawl-out behavior (means ± SE) as assessed in 5-minute intervals over the 60-minute experimental period for individuals that received either a high-risk (conspecific alarm cues, red dots and lines) or a low-risk stimulus (water control, blue dots and lines) during the experiment. The dotted line represents the timepoint at which the chemical stimulus was added to the tank.

**References**

Adams DB, Collyer M, Kaliontzopoulou A, Baken E. 2021. geomorph: Geometric morphometric analyses of 2D/3D landmark data. R package version 4.0.0.

Bates D, Mächler M, Bolker B, Walker S. 2015. Fitting linear mixed-effects models using lme4. J Stat Softw. 1:1-48.

Beaty LE, Wormington JD, Kensinger BJ, Bayley KN, Goeppner SR, Gustafson KD, Luttbeg B. 2016. Shaped by the past, acting in the present: transgenerational plasticity of anti-predatory traits. Oikos. 125:1570-1576.

Lüdecke D, Makowski D, Ben-Shachar MS, Patil I, Waggoner P, Wiernik BM, Thériault R, Arel-Bundock V, Jullum M, Bacher E. 2024. performance: assessment of regression models performance. R package version 0.12.2.

Meuthen D, Reinhold K. 2023. On the use of antibiotics in plasticity research: gastropod shells unveil a tale of caution. J Anim Ecol. 92:1055-1064.

Morton FB, Altschul D. 2019. Data reduction analyses of animal behaviour: avoiding Kaiser's criterion and adopting more robust automated methods. Anim Behav. 149:89-95.

Osborne TR, Stehman SV. 2022. Improving external shell volume estimation in snails using landmark-based size measurements. J Molluscan Stud. 88:eyac032.

Rohlf FJ. 2015. The tps series of software. Hystrix. 26:9-12.

Zelditch ML, Swiderski DL, Sheets HD, editors. 2012. Geometric morphometrics for biologists: a primer, Second Edition ed. London: Academic Press.
